# Spontaneously ruptured breast metastasis from renal cell carcinoma 20 years post-nephrectomy: a case report and literature review

**DOI:** 10.3389/fmed.2026.1859653

**Published:** 2026-08-11

**Authors:** Chengmin Huang, Yaqi Wang, Qiufeng Zhou, Qiuchao Jin

**Affiliations:** 1 Department of Surgery, Changxing People’s Hospital, Changxing, Huzhou, China; 2 Department of Pathology, Changxing People’s Hospital, Changxing, Huzhou, China; 3 Department of Ultrasound Medicine, Changxing People’s Hospital, Changxing, Huzhou, China

**Keywords:** breast metastasis, case report, metastasectomy, oligometastasis, renal cell carcinoma

## Abstract

**Background:**

Breast metastases from extramammary malignancies are rare, and those originating from renal cell carcinoma (RCC) are exceptionally uncommon. These lesions often follow an indolent course, delaying recognition until acute, life-threatening complications such as hemorrhagic rupture occur. The lack of standardized management guidelines further impedes timely intervention.

**Case presentation:**

An 88-year-old woman with a history of radical nephrectomy for clear cell RCC 20 years prior presented with active hemorrhage from a spontaneously ruptured right breast mass. Two years earlier, a 2.5 × 1.5 cm nodule (BI-RADS 3) had been detected but managed conservatively without biopsy, gradually enlarging over the following two years. On admission, physical examination revealed a large, tender mass (∼6.5 × 6 × 7.5 cm) with skin erythema, ulceration, and active bleeding. CT demonstrated the breast mass and revealed two additional imaging-suspected lesions: a right pulmonary nodule and a right adrenal mass. After hemodynamic stabilization, right simple mastectomy was performed, achieving definitive hemostasis. Histopathology and immunohistochemistry (CAIX+, PAX-8+, vimentin+; GATA-3-, GCDFP-15−, ER−, PR−) confirmed metastatic clear cell RCC. The patient declined systemic therapy. At 30-month telephone follow-up, she reported no symptomatic local recurrence or new symptoms; however, no postoperative imaging was performed to confirm this.

**Conclusion:**

This case illustrates a critical two-stage progression of breast metastasis from RCC—indolent growth followed by rapid enlargement and hemorrhagic rupture. It underscores that while a BI-RADS 3 lesion in an RCC survivor may be managed with standard short-interval imaging, any interval enlargement or morphological change should prompt a lower threshold for tissue confirmation. An IHC panel (PAX-8/CAIX positivity with negative breast-specific markers) is essential for confirming metastatic RCC. The absence of postoperative imaging in this case—with follow-up limited to symptom-based telephone assessment—highlights the critical importance of radiological surveillance to objectively evaluate disease status.

## Introduction

Metastatic breast tumors originating from extramammary malignancies are relatively rare, accounting for approximately 1% of all breast malignancies (1). Among these, renal cell carcinoma (RCC) is an extremely rare primary source, contributing to less than 1.3% of such metastases (2). RCC typically metastasizes to the lungs, bones, liver, or brain (3); breast involvement is notably infrequent. Metastatic RCC to the breast often mimics primary breast carcinoma or benign tumors, presenting as solitary, painless, well-circumscribed masses. Although progression to rupture and hemorrhage is uncommon, it carries serious diagnostic implications, especially because early symptoms are frequently absent. This poses substantial challenges for primary care clinicians and underscores the need for early detection. In the era of modern oncology, these indolent yet aggressive metastases can be understood within the concept of oligometastatic disease, which carries distinct therapeutic implications. Herein, we report a rare case of breast metastasis from RCC that was initially managed as probably benign based on imaging and required metastasectomy for hemorrhage control. We also discuss the diagnostic pitfalls and broader therapeutic considerations in oligometastatic RCC, particularly in the context of metachronous breast metastasis.

## Case presentation

An 88-year-old woman presented to the emergency department with active hemorrhage from a spontaneously ruptured mass in the lower quadrant of the right breast. On examination, her pulse was 105 beats/min, blood pressure 91/55 mmHg, and respiratory rate 22/min. A large, tender mass measuring approximately 6.5 × 6 × 7.5 cm was palpated in the same area, with overlying skin ulceration but no axillary lymphadenopathy.

**Figure 1:**
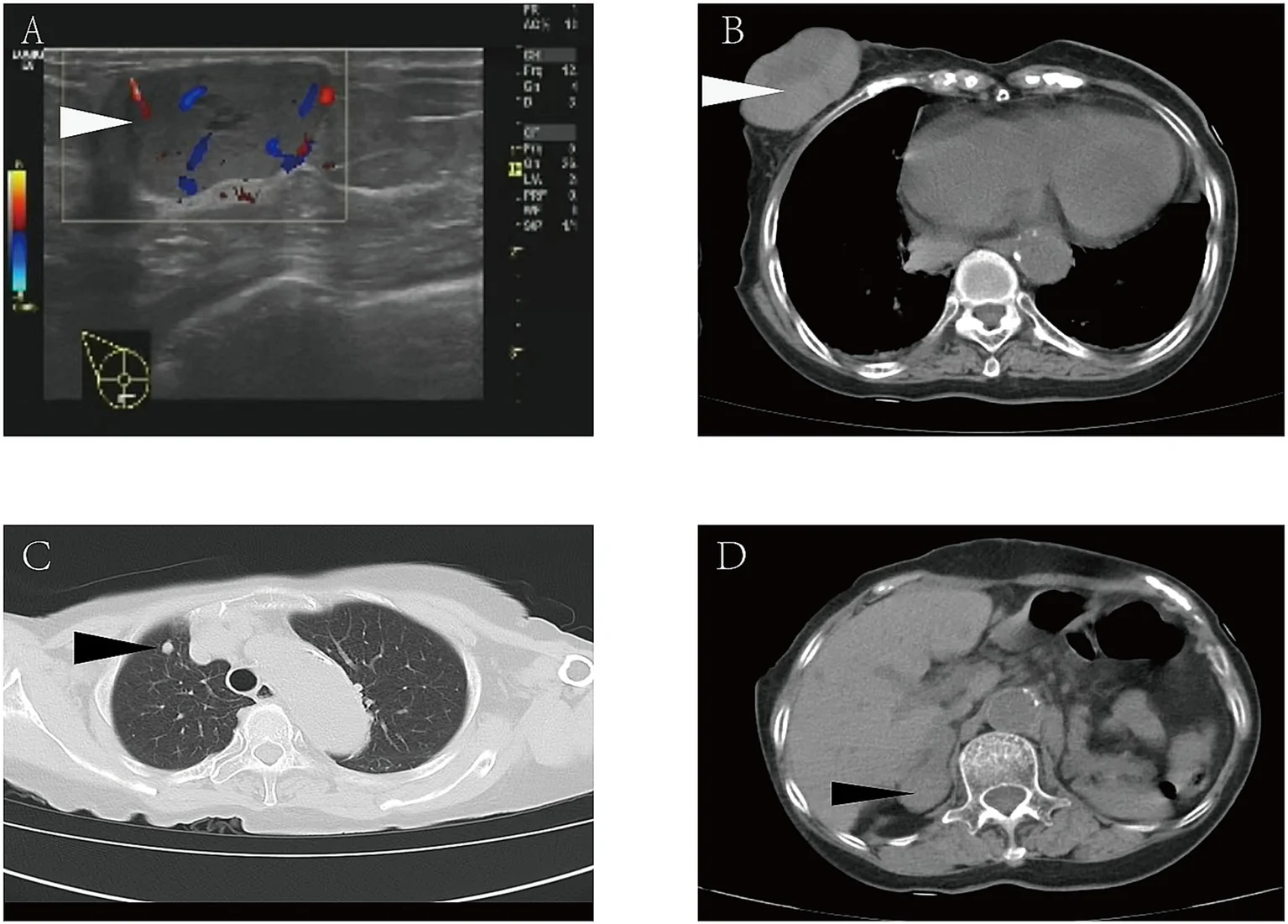
Imaging findings of the breast mass and suspected lesions (indicated by arrows). **(A)** Ultrasound 2 years before admission: 2.5 × 1.5 cm hypoechoic nodule with clear margins, BI-RADS 3. **(B)** Non-contrast CT at admission: 6.5 × 6 × 7.5 cm right breast mass with mixed solid-cystic components, suggesting intratumoral hemorrhage. **(C)** CT: Right pulmonary nodule. **(D)** CT: Right adrenal mass.

She had undergone a left radical nephrectomy for clear cell RCC at an outside hospital 20 years earlier. Due to the prolonged interval and the patient’s lack of regular postoperative follow-up, she was unable to provide any detailed information regarding the original surgery or pathology, including the primary tumor size, Fuhrman grade, pathological stage (TNM), margin status, or vascular invasion. No metastatic disease was documented at the time of initial presentation, and the patient did not receive systemic therapy postoperatively. There was no family history of breast cancer, renal cell carcinoma, or other malignancies. The patient lived with her children and was able to perform simple activities of daily living. Two years before the current presentation, she visited a community hospital for a right breast nodule. Ultrasound revealed a 2.5 × 1.5 cm hypoechoic nodule classified as BI-RADS 3 (Figure 1A), suggesting a benign lesion. Given the benign imaging features and her remote history of RCC, no biopsy was performed. During this period, the mass gradually enlarged, but neither the patient nor her family sought further evaluation. In the month before admission, the lesion rapidly enlarged, with progressive erythema, edema, and skin thinning. Spontaneous rupture occurred two hours before arrival, causing active hemorrhage and hemodynamic instability requiring emergency intervention.

On admission, the bleeding breast lesion was sutured and a compressive dressing applied for hemostasis. Laboratory tests included a complete blood count and renal/liver function tests. Chest and abdominal CT revealed the right breast mass (Figure 1B) and two additional imaging-suspected lesions: a right pulmonary nodule (approximately 1.5 cm) (Figure 1C), and a right adrenal mass (approximately 2.5 cm) (Figure 1D). The pulmonary nodule appeared as a well-defined solid lesion, and the adrenal mass presented as a homogeneous soft-tissue density. Both lesions were considered suspicious for metastases based on their imaging appearance and the patient’s known history, but no percutaneous biopsy was performed due to their asymptomatic nature and the patient’s preference. No other metastases were identified. No prior chest or abdominal CT was available for comparison, as the patient had only undergone breast ultrasound at the community hospital two years earlier. Mammography and breast MRI were not performed due to the patient’s advanced age and inability to tolerate the required positioning.

**Figure 2:**
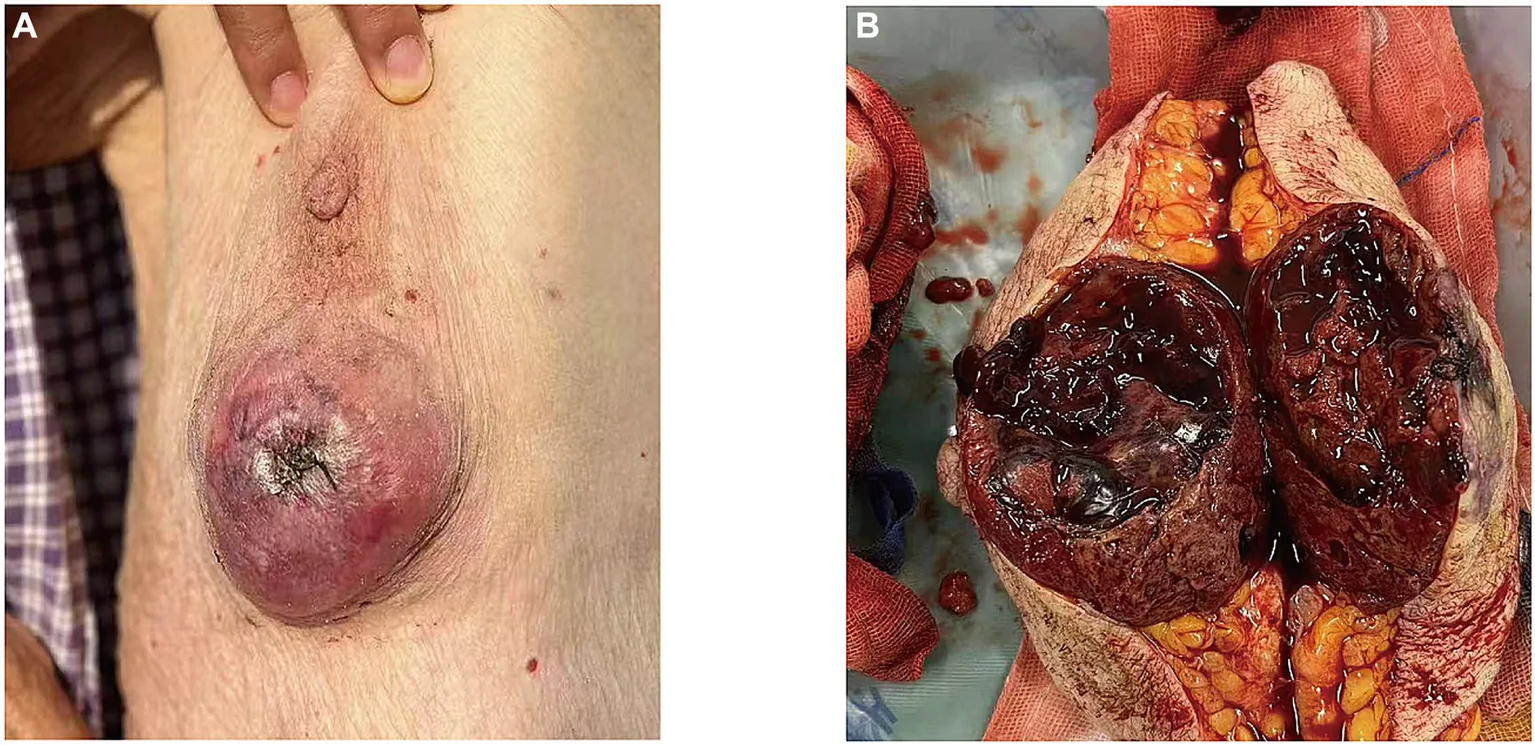
Clinical presentation and gross specimen of the ruptured breast metastasis. **(A)** One week after suture and compression (preoperative): No active bleeding, but skin erythema and edema persisted. **(B)** Gross specimen: A 6 × 6 × 7.5 cm grayish-red, friable nodule.

Right simple mastectomy was performed one week after admission to achieve definitive hemostasis and remove the infected, ulcerated mass. Preoperatively, the mass appeared as shown in Figure 2A. The resected specimen measured 14 × 10 × 8 cm and contained a 6 × 6 × 7.5 cm grayish-red, friable nodule located 2 cm from the nipple, adherent to the skin. On sectioning, the nodule revealed cystic hemorrhagic foci (Figure 2B). Intraoperative frozen section suggested metastatic carcinoma, consistent with the patient’s history. Given the intraoperative diagnosis of metastasis rather than primary breast cancer, axillary lymph node dissection was not performed.

**Figure 3:**
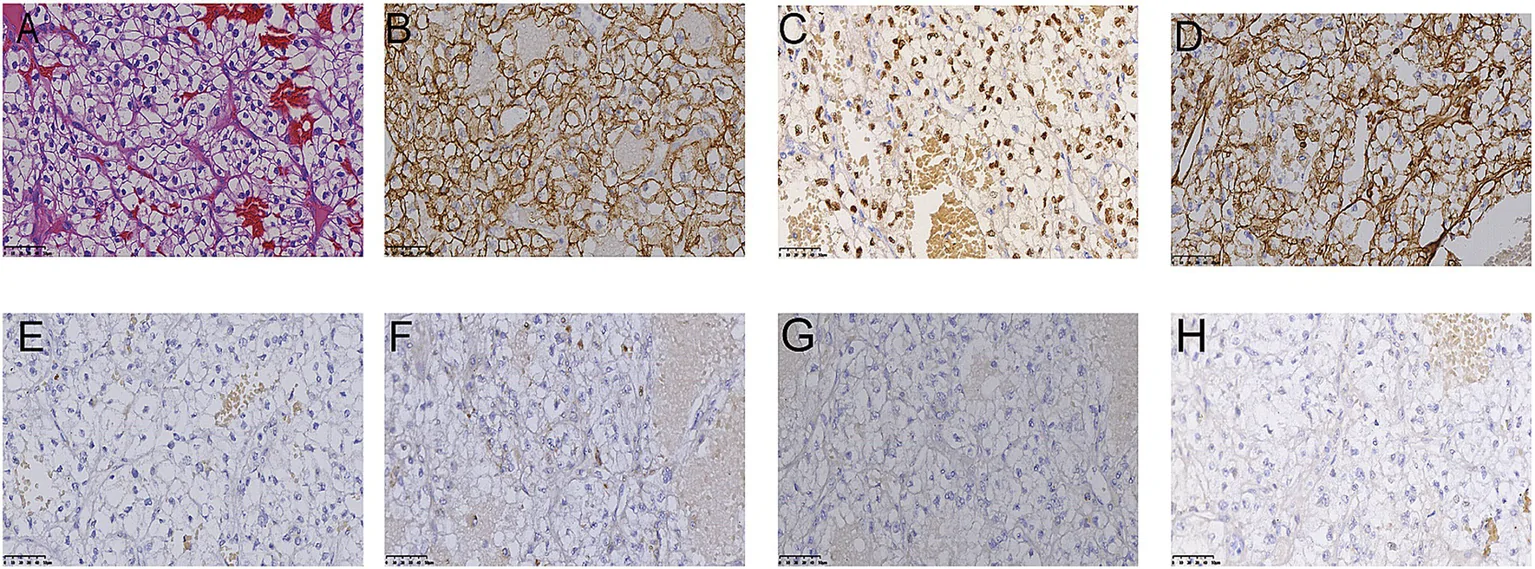
Histopathology and immunohistochemistry (all ×40). **(A)** HE: Nests of clear cells with cytoplasmic vacuolization and thin-walled vessels. **(B)** CAIX positive **(C)** PAX-8 positive. **(D)** Vimentin positive. **(E)** GATA-3 negative. **(F)** GCDFP-15 negative. **(G)** ER negative. **(H)** PR negative.

Final histopathology confirmed metastatic clear cell RCC, showing nests of clear cells with cytoplasmic vacuolization and prominent thin-walled vascular networks (Figure 3A). Immunohistochemically, tumor cells were positive for renal lineage markers (CAIX, PAX-8, vimentin; Figures 3B–D) and negative for breast-specific markers (GCDFP-15, GATA-3, ER, PR; Figures 3E–H), with a Ki-67 proliferation index of 5%. The patient declined systemic therapy. At 30-month telephone follow-up, the patient reported that no local recurrent mass or ulceration was palpable on her right chest wall, and she denied any chest tightness, cough, or lumbago-abdominal discomfort. However, it is important to emphasize that these were symptom-based assessments via telephone only, and no postoperative imaging was performed to objectively confirm the status of the pulmonary or adrenal lesions, which constitutes a major limitation in assessing true disease progression. Informed consent was obtained from the patient for publication of this case report and accompanying data. The study protocol was approved by the Ethics Committee of Changxing People’s Hospital.

## Discussion

Breast metastasis from RCC is exceedingly rare. In a population-based analysis of 11,157 patients with metastatic RCC, the most common sites were the lung (45.2%), bone (29.5%), and liver (20.3%); breast involvement was not separately reported, reflecting its exceptional rarity (3). Extramammary metastases to the breast account for only 0.5–2% of all breast malignancies, and among these, RCC is an extremely rare primary source, with fewer than 60 cases reported in the literature (4, 5). Our patient exhibited an indolent but unpredictable disease course: the initial breast mass showed benign features, leading to neglect by community physicians, and eventually progressed to spontaneous rupture with life-threatening hemorrhage, drawing clinical attention.

Metastatic RCC to the breast typically presents at an early stage as a solitary, painless, well-circumscribed mass lacking skin changes or nipple retraction, thereby mimicking benign lesions (6, 7). As a hematogenous metastasis, ultrasonography typically reveals a hypoechoic mass with well-defined margins and abundant internal vascularity on color Doppler (2, 8–10). Mammography shows a high-density mass with circumscribed borders and no associated microcalcifications or spiculations (8–10). On MRI, it often appears as a round or oval mass with hypointense on T1-weighted images and hyperintense on fat-saturated T2-weighted images, with rapid contrast enhancement and plateau or washout kinetics (11, 12). Unlike primary breast cancers, metastatic lesions generally lack spiculated margins, microcalcifications, and desmoplastic reaction. In our patient, CT showed a mixed solid-cystic mass, and the surgical specimen revealed a renal-texture tumor with hemorrhagic cavities containing clotted blood. These features are consistent with RCC metastases: clear cell RCC is highly vascular, with a delicate sinusoidal network that may predispose to spontaneous rupture, leading to intratumoral hemorrhage and cystic degeneration. Such findings are rarely seen in primary breast cancer. Diagnostic delay occurred at two levels: clinicians initially categorized the mass as probably benign due to its imaging features and the remote RCC history; later, the patient and family failed to recognize its progressive enlargement. The latter delay allowed progression to hemorrhagic rupture. This sequence of events highlights a key clinical lesson: while a BI-RADS 3 lesion in an RCC survivor does not automatically mandate biopsy and may still be managed with standard short-interval imaging, this case underscores an important caveat: any interval enlargement or morphological change should prompt a lower threshold for tissue confirmation, as the context of prior RCC can transform a “probably benign” finding into a potential metastatic warning sign.

However, imaging alone is insufficient; definitive diagnosis relies on histopathology and immunohistochemistry (IHC). Microscopically, metastatic clear cell RCC is characterized by nests of polygonal cells with clear cytoplasm and a rich sinusoidal vascular network (11). IHC is essential for definitive diagnosis: a renal lineage profile (PAX-8 +, CAIX +, vimentin +) combined with negativity for breast-specific markers (GATA-3, GCDFP-15, ER, PR) confirms the diagnosis (11, 13). PAX-8 is highly sensitive for RCC, and CAIX is expressed in over 90% of clear cell RCC cases; these markers offer superior reliability compared to CD10, which may be lost in metastatic deposits (14, 15).

From an oncological perspective, if the imaging-suspected pulmonary and adrenal lesions are indeed metastatic, this case would fit the definition of oligometastatic RCC (OM-RCC)—an intermediate state with a limited tumor burden (typically ≤5 metastases) (16, 17). OM-RCC can be categorized by timing into synchronous (metastases present at initial diagnosis) and metachronous (metastases developing after curative treatment of the primary tumor) (18, 19). Based on the presence or absence of ongoing systemic therapy, it can be further subdivided into oligoprogression (during systemic therapy) and oligorecurrence (in the absence of systemic therapy) (20). According to the ESTRO-EORTC consensus classification (18), our patient’s presentation — if the three lesions are confirmed as metastases — would meet the criteria for metachronous oligorecurrence, a subtype associated with more indolent tumor biology and favorable prognosis. However, it must be emphasized that the pulmonary and adrenal lesions were not pathologically verified; therefore, the following discussion of MDT is provided as a theoretical framework rather than a definitive characterization of this case.

For such patients, metastasis-directed therapy (MDT) has emerged as a cornerstone of management, aiming to achieve durable local control and defer systemic therapy (19–23). Accumulating evidence supports the efficacy of MDT for metachronous OM-RCC. A large retrospective study of systemic therapy-naïve patients revealed a 5-year systemic therapy-free survival (STFS) rate of 44.9% after MDT, with a 2-year progression-free survival (PFS) of 63.6% in favorable-risk patients (21). A risk model based on DFI (disease-free interval) < 1 year and ≥2 lesions helps stratify patients accordingly.

Three MDT modalities are available.

### Surgical metastasectomy

Complete surgical metastasectomy reduces mortality risk (HR = 0.54), whereas incomplete resection confers no survival advantage — a finding confirmed by a meta-analysis (HR = 2.15 for no surgery vs. metastasectomy) (24, 25). Metastasectomy is most commonly considered for metastases to the lung, pancreas, adrenal gland, isolated lymph nodes, or local recurrence, particularly when complete resection is achievable. Favorable selection factors include solitary metastasis, pulmonary location, metachronous presentation (disease-free interval >12 months), and IMDC favorable/intermediate risk (19).

### Stereotactic body radiotherapy (SBRT)

For non-surgical candidates, SBRT achieves >90% local control for intracranial, bone, lung, adrenal, and pancreatic oligometastases (26–29). The SABR-ORCA meta-analysis reported a 1-year local control rate of 89% and grade 3–4 toxicity below 1% (26, 30). Moreover, a 2025 meta-analysis of 29 studies found that SBRT alone yielded a 1- to 2-year systemic therapy-free survival of 87.0% (95% CI 76.2–95.2%) in oligometastatic RCC, highlighting its potential to defer systemic therapy (23).

### Percutaneous ablation

For patients with oligometastatic renal cell carcinoma who are frail or at high surgical risk, percutaneous ablation (e.g., cryoablation, radiofrequency ablation [RFA], microwave ablation) is recognized by NCCN clinical practice guidelines as a viable treatment option (31). In the oligometastatic setting, retrospective studies have reported 5-year overall survival (OS) rates ranging from 50 to 100% after RFA, with a low risk of non-pneumothorax complications (1.2%) and mortality (0.1%) (32, 33).

In our case, the patient presented with a 6.5 cm breast mass with active hemorrhage and skin ulceration. On admission, the bleeding lesion was sutured and a compressive dressing applied to achieve temporary hemostasis. Given the life-threatening hemorrhage and local infection, definitive surgical metastasectomy was performed one week later after stabilization, as SBRT or RFA would have required tumor stabilization and posed risks of radiation-induced skin necrosis or delayed bleeding. Applying the risk model, her long DFI (20 years) and the fact that only the breast lesion was symptomatic (the lung and adrenal lesions were asymptomatic) classified her as favorable-risk for MDT of that lesion, supporting the decision for MDT without immediate systemic therapy. The two asymptomatic lesions were small. Given the patient’s advanced age and refusal of systemic therapy, active surveillance was recommended (with sequential SBRT if progression occurs). However, due to her age and travel distance, she did not attend scheduled postoperative imaging visits. This symptom-driven approach was a shared decision based on patient preference rather than a validated standard. Notably, without imaging, we cannot confirm the stability of the asymptomatic lesions; thus, our experience offers no robust evidence for this strategy over structured surveillance.

Several limitations and unanswered questions should be acknowledged. As a single case report, this study lacks generalizability. The patient declined systemic therapy and further MDT on the pulmonary and adrenal lesions, precluding evaluation of whether complete MDT could have delayed systemic therapy or improved outcomes, as well as the potential role of adjuvant immunotherapy (e.g., pembrolizumab) for M1NED status. Most importantly, no postoperative imaging was performed because the patient, due to advanced age and long travel distance, did not attend scheduled visits; only telephone follow-up was available, which confirmed good general condition but cannot reliably exclude local recurrence or progression of the asymptomatic metastases. This absence of radiological surveillance significantly weakens the evidence supporting the “active surveillance” strategy described above. Additionally, the optimal surveillance interval for asymptomatic oligometastases in elderly patients remains undefined. Prospective studies or registry data are needed to validate the “symptom-priority, on-demand MDT” strategy. Furthermore, our discussion of oligometastatic disease and metastasis-directed therapy should be interpreted with caution, as it is based on imaging-suspected rather than pathologically confirmed metastases.

## Patient perspective

The patient was satisfied with the mastectomy, which relieved pain and bleeding, greatly improving her quality of life. She preferred community follow-up and agreed to return only if new symptoms developed.

## Conclusion

Breast metastasis from RCC is insidious and often mimics benign lesions. In RCC survivors, while standard imaging follow-up remains appropriate for stable BI-RADS 3 lesions, any interval growth should prompt tissue confirmation. For elderly patients, a quality-of-life-oriented approach may be considered, ideally with structured imaging surveillance. Our case underscores that radiological follow-up is essential to validate any surveillance strategy, as its absence limits the generalizability of our experience.

## Data Availability

The original contributions presented in the study are included in the article/Supplementary material, further inquiries can be directed to the corresponding author.
